# The Clinical Use of Prisms

**Published:** 1889-09

**Authors:** 


*The Clinical Use of Prisms.* By ERNEST E. MADDOX, M.B. Bristol: JOHN WRIGHT & CO. 1889.

This little book is likely to be of considerable use in assisting in the elucidation of a difficult subject.

It first deals with the physical effects of prisms upon light, and then describes how the change in direction of the rays of light, which the prism produces, can be applied to the diagnosis or treatment of diplopia, or muscular insufficiency.

The author has evidently given much attention to the subject; and his book, though simple, is complete and good.

There is an excellent chapter upon the decentering of lenses—a matter of more clinical importance, in our opinion, than prescribing prisms themselves; and the chapter on the analysis of the prismatic effect of spectacles deals with the centering of their lenses, and the value as prisms of convex and concave lenses of various strengths.

The author recommends the use of prisms in various ways for purposes of diagnosis. Thus he recommends their use: (1) as a measure of the absolute minimum or maximum of convergence, or of its relative range; (2) to dissociate convergence and accommodation; (3) to decide the presence of binocular vision; (4) to disclose any tendency to vertical diplopia. They are also recommended to be worn by the patient as a method of treatment: (1) to relieve the strain of convergence; (2) to overcome curable diplopia by orthoptic exercises; (3) to correctan incurable diplopia; and (4) to relieve vertical diplopia.

We commend the book to the consideration of any who are engaged in ophthalmic work, and to those who are interested in the physical aspects of optics.

